# Student Nurses’ Perceptions of the Role of High-Fidelity Simulation in Developing Decision-Making Skills for Clinical Practice: A Qualitative Research Study

**DOI:** 10.1177/23779608241255299

**Published:** 2024-05-20

**Authors:** Naim Abdulmohdi, Andrew McVicar

**Affiliations:** 1 School of Nursing and Midwifery, Faculty of Health, Education, Medicine and Social Care, 2369 Anglia Ruskin University, Cambridge, UK; 2 School of Nursing and Midwifery, Faculty of Health, Education, Medicine and Social Care, 152391 Anglia Ruskin University, Chelmsford, UK

**Keywords:** nursing students, other-zero level, qualitative research, research, nursing education

## Abstract

**Introduction:**

The integration of high-fidelity simulation (HFS) in nursing education has increased, but its effect on students’ clinical decision-making skills and their ability to transfer these skills to clinical practice remains unclear.

**Aim:**

This qualitative study aimed to explore nursing students’ perceptions of simulation's role in developing decision-making skills for clinical practice.

**Methods:**

Twenty-three self-selected final-year nursing students participated in an HFS exercise in 2016. They engaged in “think-aloud” activities during the simulation, reviewed videos of their performance, and attended a structured debriefing session. Four to six weeks later, face-to-face semistructured interviews were conducted to gather their views on the application of learning from simulation into practice. Thematic analysis was used to analyze the interview data.

**Results:**

Four themes emerged from the analysis: “enhancing clinical decision-making skills,” “recognition of the types of clinical decision-making,” “recognition of cognitive biases,” and “transferability and integrating theory into practice.” Simulation improved student self-awareness, decision-making skills, and recognition of cognitive biases applied in practice. Overall, students found that the simulation improved their ability to apply theoretical knowledge gained through simulation to practice. The students’ perception of the authenticity of activities in relation to real-world scenarios played a crucial role in enhancing the transferability and application of acquired knowledge from simulation to clinical practice.

**Conclusion:**

The findings provide valuable insights into how simulation optimizes learning and decision-making skills, ultimately promoting effective care in clinical settings.

## Introduction

Over the past 30 years, there has been an increased focus on patient safety and the recognition of the impact of human errors in healthcare (Urquhart et al., 2021). Failure to identify crucial clinical data and subsequent failure to make suitable clinical judgments were significant factors contributing to the failure to effectively respond to rapidly deteriorating patients (National Confidential Enquiry into Patient Outcome and Death, 2012). There is a worldwide shortage of nurses, and the limited availability of appropriate clinical placement is adding more pressure on higher education institutions and placement providers to explore how to ensure nursing students have “fitness to practice” at the point of registration (National Council of State Boards of Nursing, 2022; Royal College of Nursing, 2018). Simulation-based education (SBE) is frequently utilized in nursing curricula, but there are significant variations in the practice hours required by nursing courses across the world (Garrow et al., 2022) and how SBE is integrated into the nursing curricula. Nursing programs are under significant scrutiny to produce graduates with the ability to provide safe and effective care highlighting the demand for additional research to substantiate evidence-based simulation practices in education.

One of the aims of nursing education and professional bodies is to ensure that the learning activities in nursing curricula are not only focused on the theoretical attainment of knowledge, attitude, and skills but also learning that can be translated and effectively integrated into the real world of nursing practice. Moreover, it is vital to ensure that the learning activities adequately prepare students to be competent practitioners in responding effectively to deteriorating patients and able to protect patient safety. Given the necessity for nurses to regularly make decisions regarding patient care, it is crucial to investigate how student nurses acquire and subsequently apply decision-making skills in their practice as they progress toward becoming qualified nurses. High-fidelity simulation (HFS) provides healthcare educators with opportunities to refine a learner's decision-making skills. Understanding whether simulation plays a role in enhancing the transfer of learning decision-making skills to practice may encourage educators to integrate simulation more frequently into their teaching methods. However, there is limited qualitative research exploring nursing students’ experiences regarding the transferability of learning from simulation to clinical practice. This study provides a qualitative evaluation of the perceived benefits of HFS in learning clinical decision-making skills and their practical application in clinical settings.

## Review of Literature

In the last 2 decades, clinical decision-making has been extensively studied, primarily in natural settings with expert nurses using qualitative methodology (Smith, 2013), and more recently in simulation settings using experimental quantitative designs (Cobbett & Snelgrove, 2016; Woda et al., 2017). Nursing research in natural settings either focused on the analytical or nonanalytical theories of decision-making with limited exploration of a pluralist approach to decision-making. The effect of HFS on clinical reasoning and nursing education produced inconclusive findings in previous quantitative research (Cant & Cooper, 2017; Mok et al., 2016; Theobald et al., 2021). While there is supporting evidence indicating that HFS positively impacts students’ learning, several studies did not find significant improvement in the effect of HFS on critical thinking scores (Knoesel, 2017; Son, 2020; Wood & Toronto, 2012), perceived self-confidence (Merriman et al., 2014; Woda et al., 2017; Young & Jung, 2015) and clinical decision-making (Cobbett & Snelgrove-Clarke, 2016). Other studies though identified that HFS seems to have more consistent positive and significant effects on clinical performance (Lee et al., 2016; Merriman et al. 2014; Reid et al., 2020). Cant and Cooper (2017) reported that the inconsistency of results might be due to a lack of high-quality study designs and the use of a constellation of instruments. There is a lack of research in the field of simulation that is based on the theory of clinical decision-making. The dual process theory (DPT) in clinical decision-making outlines two distinct types: Type 1, characterized by automaticity and intuition, quick, impulsive often influenced by experience and more susceptible to cognitive biases. It includes pattern recognition and rapid decision-making. Type 2 is analytical, slow, deliberate, methodological and reflective, relying on the hypotheticodeductive reasoning approach (e.g., inductive and deductive reasoning; Croskerry & Nimmo, 2011). The DPT provides a pluralistic and integrated approach that considers both types of CDM that other theories in CDM do not offer. DPT is also based on the consensus of experts in the field of cognitive psychology with neurological evidence to support it (Evans & Stanovich, 2013).

Cognitive biases are inherent tendencies in information processing, influenced by individuals’ experiences and beliefs. Tversky and Kahneman (1974) identified three common biases in human decision-making: representativeness, availability, and anchoring. Croskerry et al. (2013a) extensively studied biases in acute and emergency medicine; however, there is limited nursing research in this field. Brannon and Carson (2003) identified the use of representativeness among nursing staff that negatively affected decisions (e.g., triage decision, concern escalation). Riva et al. (2011) reported the impact of anchoring bias on clinicians’ pain judgments. Al-Moteri et al. (2020) found that 63% of nurses used confirmation bias, resulting in ineffective decision-making when working with deteriorating patients. Recent reviews indicate that cognitive biases can affect patient assessment, diagnosis, and treatment, but the research is largely focused on medical staff decisions (Jala et al., 2023; Thirsk et al., 2022).

More importantly, there is limited research that explores the translation of learning clinical decision-making skills from HFS to clinical practice despite the wide use of HFS in nursing education. In previous qualitative research, students perceived HFS to support the recognition of acutely ill patients (Hastad et al., 2019; Liaw et al., 2012), enhance deliberate practice (Botma, 2014), promote active learning (Loke et al., 2014) and improve students’ confidence (Kaddoura et al., 2016). Unfortunately, there is a paucity of evidence as to which teaching strategies are effective in promoting the transfer of learning to clinical practice and whether learning CDM skills using HFS can be transferred to clinical nursing practice. Hanshaw and Dickerson (2020) reported more research is required to assess the transference of simulated learning into clinical nursing practice. El Hussein and Concannon (2022) identified that most of the reviewed studies focused on short-term transfer of learning to practice with limited follow up. This study examines how student nurses perceived the value of what they have learnt about clinical decision-making through HFSs and its impact on their clinical practice.

Transferring the learning into practice is a complex process that is affected by multiple factors such as students’ abilities and motivation, educational designs and the working environment (Ewertsson et al., 2015; Fathi & Ibrahim, 2023). It also requires students to internalize the newly developed knowledge with existing knowledge. The degree and nature of what can be effectively applied from simulated environments to real-world situations remain uncertain, especially when considering intricate processes like clinical decision-making. Transferring learning to a different context necessitates conscious cognitive effort, including reflective thinking and the purposeful extraction of key problem attributes (Johnston et al., 2019). The similarity between simulation settings and clinical environments supports pattern recognition, facilitating the application of simulated learning to clinical practice (Liaw et al., 2012). Nash and Harvey (2017) found that if students perceive significant differences between simulated activities and real-world situations, it can negatively impact the transfer and practical application of what they have learnt. Simulation recognizes the importance of learning through experience and reflection on experience. Tailoring the learning experience during a performance, encouraging abstract thinking and generalization during debriefing, and providing explanations help learners reflect on their actions and gain insights that extend beyond specific scenarios, applying them to real-world clinical practice (Kolb, 2015). This is critical to support students’ ability to transfer learning to the real world of practice (Botma, 2014). Understanding the benefits of simulation experiences based on a decision-making theory and how this learning translates into clinical practice can provide guidance to nurse educators for improving simulation design, ultimately enhancing clinical applications and patient care.

## Method

### Aim of the Study

The aim of this study was to explore nursing students’ perceptions of simulation's role in developing decision-making skills for clinical practice. The main research question for this study was:

1. How do students perceive the usefulness of high-fidelity simulation experience in learning clinical decision-making skills for their clinical practice?

### Study Design

This study adopted a qualitative descriptive research design. This research was part of a multiphase research study that had two phases. The participants attended an individualized simulated session using a high-fidelity simulation mannequin (SimMan Essential 3G^TM^) at the university clinical laboratory in phase one of our study (Abdulmohdi & McVicar, 2022). The current article reports the findings from the second phase of our study, using a qualitative descriptive research approach.

### Participants and Recruitments

A purposive sampling technique was employed to recruit a cohort of self-selected nursing students who voluntarily participated in this study in 2016. According to the inclusion criteria, participants were final-year nursing students enrolled in a bachelor of science program at one university in England, had previous experience and were familiar with mannequin-based HFS.

An invitation was sent to all students in the cohort via their students’ email and in a face-to-face session with the course leader. During those weeks between the simulation experience and the follow-up interview, all participants were in a full-time clinical placement in their final year of the course. The first author, a senior lecturer in nursing, was only involved in teaching the participants during their second year and the second author, a professor in health science, was not directly involved in teaching the participants.

### Data Collection

Four to six weeks after students attended a simulation experience (described in Table 1) using HFS (SimMan Essential 3GTM) in Phase 1 (Abdulmohdi & McVicar, 2022), a follow-up face-to-face semistructured interview was conducted with each student in this phase. The interview lasted 10–20 min and was audiorecorded with the participants’ permission. Two out of the 23 interviews lasted less than 15 min, and the total time for all 23 interviews was approximately 405 min.

**Table 1.** Stages of the Simulation.

Stages of simulation	Content	Duration (minutes)
1. Prebriefing	Orientation students were reminded of the airway, breathing, circulation, disability and exposure approach.	10–15
2. Performance	Students were asked to think aloud and respond to patient's needs.	15–20
3. Debriefing	Structured debriefing tool was used (Ahmed et al., 2013)	15–25
4. Debiasing	The facilitator introduced students to the types of clinical decision-making and cognitive biases and provided them with a list of biases and debiasing strategies to reflect on future clinical practice (Croskerry et al., 2013a)	10–15

The interview took place in quiet prebooked rooms at the university campus when the students were coming for separate training and were available to attend. Prior to the interview, the content of the interview was discussed using a participant information sheet (PIS). It clarified my role as a researcher and the participant's consent for the study was obtained. The first author conducted all the interviews, took notes, and audiorecorded each interview. An interview guide was reviewed by the research team, piloted with the first two participants, and no changes were deemed necessary. The guide included the following open-ended questions, which were used to explore students’ experiences of the simulation and how it influenced their practice. Consolidated Criteria for Reporting Qualitative research checklist was used to report the study results.

The opening question was: *Can you tell me how did you find the simulation experience?* Followed by *What aspect/s of the simulation experience did you find most useful to your practice? Why did you find this aspect as the most interesting? How did the simulation experience influence your practice? How did the simulation experience impact the way you make decision in practice? Tell us more about the types of decision-making you predominately use in clinical practice? How do you make decisions about your patient in clinical practice? What errors did you notice yourself making in clinical practice? Do you have examples of clinical situation, where a bias may have impacted your decision-making? Anything else do you want to add?*

### Data Analysis

To explore the content of the interviews, an inductive data-driven coding method was undertaken to generate themes within the data (Miles et al., 2014). The interviews were transcribed and submitted to thematic analysis by following the steps recommended by Braun and Clarke (2006). Firstly, all transcripts were read in one session to develop a better sense of the data and notes were written from the initial reading. After that, all the transcripts were then organized and imported into NVivo 11 software (QSR International, 2017) to manage the coding process and conduct the thematic analysis. This was an iterative process undertaken a number of times to increase the validity of the coding. All transcripts were coded in a systematic way generating a list of descriptive codes. The codes were then clustered together to identify meaningful patterns which resulted in the initial subthemes. The thematic “tree” of all the subthemes was aggregated to illustrate a smaller number of meaningful patterns that were considered the main themes. Each major theme was linked to subthemes, the descriptors, and the coded sections from the different transcripts. The analysis focused on emergent themes which addressed the primary research question of this study.

### Rigor

Several approaches were used to enhance the trustworthiness of the data (Nowell et al., 2017). The credibility of the findings was reinforced by gathering data on student nurses’ perceptions of how HFS can impact the development of decision-making skills for clinical practice. The study's research question was effectively addressed through generated data, showcasing the credibility of the findings and ensuring methodological consistency. Dependability was reinforced by drawing on supporting and contrasting literature during the discussion of results and the findings from this phase were related to results from the first phase of the study. The same researcher (NA) conducted the data collection, transcription, and analysis, adding credibility to the results. The auditability of the data was maintained by transparently describing the steps of data analysis, categories, and themes with illustrative examples. To further support the internal validity of the research and our conclusions, data analysis was independently conducted, and an agreement was reached regarding the identified themes through regular analytic sessions with the research team. An external auditor who is an experienced qualitative researcher reviewed the coding book, provided feedback and agreed on the generated themes.

### Ethical Considerations

Participation in this study was voluntary and all participants were given a PIS and supplied written informed consent before data collection began. Participants were assured that they would not be identifiable in any dissemination medium.

The School of Nursing and Midwifery Research Ethics Panel approved this study (approval number SNM/DREP/14–014) and course leaders/senior managers at the university gave institutional approval.

## Results

### Sample Characteristics

Twenty-three students participated in this study. Table 2 indicates that 87% of the participants were female, less than 30 years of age (73.9%), and primarily had limited experience (61%) in healthcare before commencing the nursing degree. A majority of the participants (47.8%) did not undergo clinical placements in the emergency department and critical care units.

**Table 2.** Sample Characteristics.

Characteristics		Number (%)
**Gender**	Female	20 (87)
	Male	3 (13)
**Previous healthcare experience**	No experience to < 2 years	14 (61)
	≥ 2 to < 4 years	3 (13)
	≥ 4 years	6 (26)
**Type of clinical placement**	Mixed (medical and surgical) without ICU or emergency unit	11 (47.8)
	Mixed (medical and surgical) with emergency unit	6 (26.1)
	Mixed (medical and surgical) with ICU	4 (17.4)
	Mixed (medical and surgical) with emergency unit and ICU	2 (8.7)
**Age range (years)**	20–25	12 (52.2)
	26–30	5 (21.7)
	31–35	1 (4.3)
	36–40	3 (13)
	>41	2 (8.7)

ICU: intensive care unit.

### Study Themes

**Figure 1.**
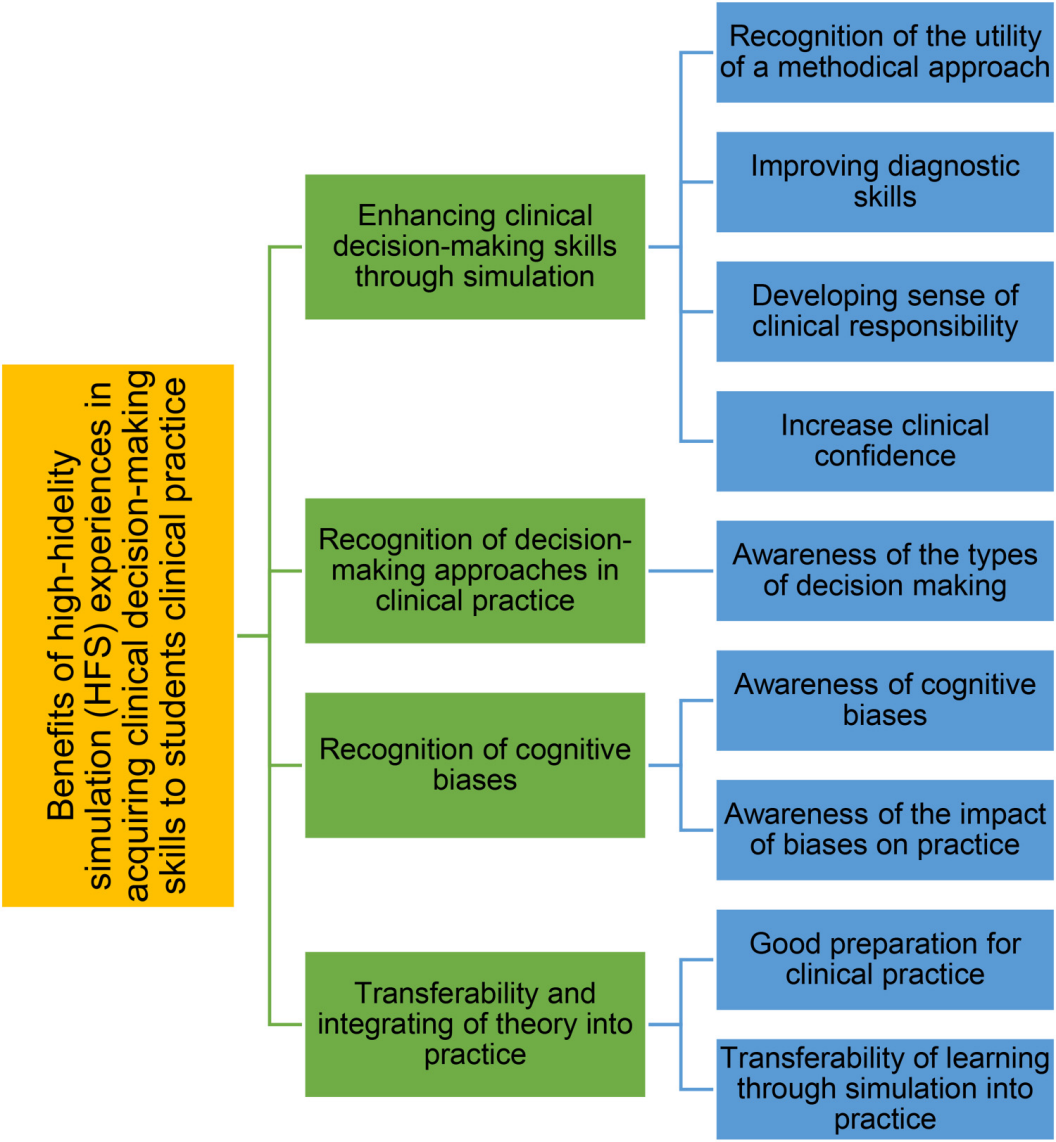
Qualitative themes and subthemes.

Twenty-three student nurses took part in the study. The interview analyses delineated themes that described the usefulness of simulation experience in enhancing students’ decision-making skills and its benefits on students’ clinical practice. Four major themes emerged from the analyses, including enhancing clinical decision-making skills, recognition of the types of clinical decision-making, awareness of cognitive biases, and the transferability and integration of theory into practice (Figure 1).

#### Theme 1—Enhancing Clinical Decision-Making Skills Through Simulation

##### Recognition of the Utility of a Methodical Approach

Students mentioned that the simulation learning experience was stimulating and engaging. They regularly discussed how they used the experience to assess their effectiveness in managing clinical situations. Students described their approach to decision-making during the simulation experience as fragmented and how it affected the outcome of their decision. The simulation increased students’ awareness of the importance of being methodical in assessing and responding to patient needs, critically evaluating their performance, and identifying areas for improvement in clinical practice.

*It made me aware that if I do things step by step it helps a lot because when I missed key information it affected the overall problem identification of that patient.* (P6).

*I think if you go through that [patient assessment and management] methodically or systematically rather, then I think it helps you identify what possible issues there are and help practice more effectively.* (P4).

##### Improving Diagnostic Skills

Similarly, students found that the simulation experience enhanced their diagnostic skills as they started to think of different alternatives before reaching conclusions and discussed how the simulation increased their awareness of the effect of cognitive errors on their diagnostic skills. The experience helped them to consider alternative explanations, think thoroughly about the true value of the clinical cues and not rushing to a conclusion.

*You need that in clinical practice because you can’t just go in one angle, there can be so many things affecting the patient, so you need to take a holistic view[..] If you can orientate the best way to care and treat patients, then they will come out at a better level and a better standard and receive better care.* (P20).

##### Developing Sense of Clinical Responsibility

Students considered that performing the task alone helped them to focus on solving the clinical problems and evaluating their performance. They described how they became more actively involved in the learning process and how being more involved helped them to understand their strengths and weaknesses. Students felt the simulation experience was like a real situation that stimulated a sense of clinical responsibility*.* They commented on remembering the simulation experience during their clinical practice. This also indicates the clinical application of learning from the simulated scenario and could suggest that students’ active involvement in the learning process may have lasting effects on knowledge and skills retention.

*It also put me on the spot, as if it was a real clinical situation and what I should do in practice […] really useful. I think it's really stayed with me.* (P4).

##### Increase Clinical Confidence

Students also discussed how they became more effective and confident in the way they process clinical information and make decisions after their simulation experience.

*I think, for me, it's given me a bit more confidence that I've got somewhere to start, it calmed me down.* (P4)

*My mentor has even commented that he's seen myself confidence-wise, feeling more able to take care of the patients.* (P17)

#### Theme 2: Recognition of Decision-Making Approaches in Clinical Practice

##### Awareness of the Types of Decision-Making

This theme was identified based on students’ statements describing the different approaches they used for their decisions and problem-solving in clinical practice. Students reported the features of the two types of decisions in their own clinical practice. They recognized the tendency to rely on quick and impulsive decisions (Type 1) based on an easy grasp of the situation and became more self-aware of the need to mitigate this limitation by seeking more information before making decisions. Further, they acknowledged that relying solely on “quick decision” is not always effective and emphasized the importance of thoughtful consideration.

*You sort of grasp the easy explanation. They are post-operative patients so hypotension must be due to bleeding. You kind go to the simple explanation and I became more aware of it [ways of thinking] since I did the simulation.* (P23)

*On many occasions, my experience has shown me that my first impression isn’t always right, it's like an instinct one, and I need to think more carefully about other things before reaching conclusions about patients’ conditions.* (P19).

Other students described a similar behavior as something they develop through routine practice when solving familiar problems and performing repetitive actions, which eventually become automatic. However, when faced with an unfamiliar encounter, they give it more attention and consciously think about it. Some students also described relying on patterns and gut feelings in their approaches to clinical situations.

*It is very easy to fall into a routine. So, if you continue the routine, I will say a lot of that is unconscious decisions, but actually, if I saw a new patient, then all of my decisions become consciously different and I’m aware of different things.* (P20)

*I think sometimes I do tend to go with my gut feeling on things.* (P6)

Students noted that the effectiveness of different decision-making approaches (types) depends on the task at hand and highlighted the benefits of being conscious and attentive in decision-making. However, they also recognized the practical challenges in applying these approaches in time-sensitive environments, acknowledging the need for quick decisions while considering the tradeoff between speed and thoughtfulness in the decision-making process. Student P7 describes a characteristic of Type 1 as a subconscious decision and explores its effectiveness from their perspective.

*My decisions are not that effective when I don’t really pay attention. It was really useful to know that the kind of decisions I make are not always the most thorough. (P7)*

*I think it is more effective being conscious [type 2] but obviously there is the environment that you need to make these decisions very quickly. So, at the moment it [ type 2] is useful but a slower process and it slows me down when I am making working in an environment that quick decision is needed.* (P12)

#### Theme 3—Recognition of Cognitive Biases

##### Awareness of Cognitive Biases

This theme identified from students’ discussions regarding their awareness of cognitive biases (such as jumping to conclusions and premature closer anchoring) in their approach to patient care, both in the simulation setting and in their clinical practice. Students acknowledged their previous ineffective strategies, such as taking shortcuts in gathering information and reaching premature conclusions without careful examination of the clinical situation.

*Jumping to conclusions for me has been a problem […] being more aware of it, now I am better at it. Once you know—I do not think you need to remember all of them* (list of cognitive biases)*. You need to know which ones are relevant to you.* (P1)

Other students reported recognizing being fixated on one issue and sometimes the clinical context led them or their colleagues to be fixated on one aspect. These comments demonstrated students’ awareness of different types of biases that could affect hypothesis generation or their actions such as premature closure and anchoring.

*I remember I homed in one possibility and forgot all other possible scenarios.* (P4) *Because it* (patient's condition) *was in a respiratory context everybody was overlooking the cardiac condition. So, they were like fixated or reaching conclusions early* (…) *I have seen it (cognitive bias) a few times in practice I became more aware of it since we went through the scenario.* (P23)

##### Awareness of the Impact of Biases on Practice

Other students recognized errors that affected patient diagnosis such as students reporting the bias “availability” (making decisions based on limited but immediately available information) in their comments from practice.

*The history said something about an MI (myocardial infarction), and the patient was a smoker, so straightaway I was thinking, ‘chest pain.’ So, I immediately went down cardiac route based on the background information. (P2)*

Most of the students described how reflecting on their experience was the most useful part of the simulation as it helped them to make sense of what they did and how to improve their skills. Students described the significance of reviewing their performance through video recordings, which provided a different perspective and allowed for critical analysis and self-observation. Reviewing the video during their reflection appeared to give students a different perspective on how they really performed rather than how they thought they performed.

*I found reviewing afterwards, so we watched the recording afterwards* (…) *you see what you’ve done and then you can reflect on that and look to yourself, which was really important.* (P20)

Overall, the theme highlights students’ growing awareness of cognitive biases and the importance of reflection in mitigating their impact on clinical practice.

#### Theme 4—Transferability and Integrating of Theory into Practice

Students’ reflections on the value of simulation experience in bridging the gap between theory learning and its application in real world of practice.

##### Good Preparation for Clinical Practice

One aspect of this theme highlighted how the simulation experience provided students with a realistic representation of the complexities they would encounter in clinical practice. They noted the similarity between the simulated scenarios and the multifaceted nature of real patients, who often present with multiple comorbidities. Students recognized that the simulation prepared them to handle the challenges of addressing various aspects of patient care simultaneously, which they found highly applicable to real-world practice.

*We were looking at multiple things happening with the same patient. Which is more applicable to practice* (…) *Obviously, in Module 7 we are focusing on one thing at a time so we can understand it properly but in the real world, patients come with multiple co-morbidities and for that, I found this simulation really helpful*.

This also agrees with student (P7) comments on the similarity of clinical pressure produced in the simulated scenario to the one they feel in clinical practice.

*I think I kept skipping steps because I was just trying to get through it. Pressure-wise, I’d say it was (simulation) quite like real patients.*

##### Transferability of Learning Through Simulation into Practice

Students reflected on how this experience enhanced the application of theory to practice, leading to improvements and changes in their clinical practice. They acknowledged the role of simulation in enhancing their application of theoretical frameworks, such as the airway, breathing, circulation, disability and exposure (ABCDE) approach in real-life situations.

*It made me appreciate the difference between having the theory and then putting it into practice. Having said that the simulation scenario was a good opportunity to improve the way I practise and take action.* (P4)

The simulation experience enables students to apply theoretical knowledge, refine their skills, and gain a deeper understanding of the practical implications of their decision-making processes, ultimately preparing them for the challenges they will face in their future clinical practice.

## Discussion

The findings of this qualitative study reflected how these 23 nursing students valued the usefulness of learning through clinical simulation and how this experience impacted their clinical decision-making and clinical applications. In this study, the simulation experience led students to immerse themselves in the problem-solving exercise as they actively sought cues to solve the clinical problem, making the simulation activity engaging and worthwhile for them. Students felt the simulation experience was like a real situation that stimulated a sense of clinical responsibility and that they needed to effectively manage the clinical situation. The proximity of the simulated experience to reality has been perceived as a crucial part of the exercise that enhanced students transferring what they learnt through simulation to their clinical practice. These results corroborate similar findings from Botma (2014), Loke et al. (2014), and Hustad et al. (2019). Botma (2014) explored the perceived benefits of three HFS sessions among eight nursing students and found that the students perceived HFS to enhance the development of deliberate practice, as well as the application and integration of theory into practice. Students in this study also described how a one-to-one session helped them focus on processing the clinical cues and making sense of the clinical situation without interruption or distraction. Interruption by others has been linked to negatively affecting nurses’ ability to think clearly or logically that have the potential to affect patient safety (Hayes et al., 2017).

Students regularly described how they used the simulation learning experience to assess their effectiveness in managing clinical situations. Their self-evaluation was useful to regulate their behaviors, measure their perceived level of competency or to determine their self-efficacy. Students focused more on their mistakes which if they did not receive debriefing or developmental feedback could have potentially resulted in negative effects on their clinical confidence. This is consistent with Hustad et al. (2019) who reported that students were over-critical of their performance in their study and the debriefing session was constructive and supportive of their learning. Dreifuerst (2012) reported that a structured debriefing and reflection on the simulation experience were critical factors to enhance nursing students’ learning, confidence, and clinical reasoning. Sedgwick et al. (2014) also found that simulation and reflective debriefing enhanced the quality of novice and experts’ clinical decision-making skills.

Students’ comments indicate that the simulation affected how they assessed and managed patients in clinical practice by aiming to enhance the content and structure of their assessment. Students’ comments here could be related to them developing two cognitive skills. The systematic way of thinking about clinical encounters is a description of lateral thinking and the ability to evaluate and relate the identified cues which can be linked to critical thinking. Both lateral and critical thinking are key components to developing effective decision-making skills (de Bono, 1970). These results concur with quantitative research findings (Dreifuerst, 2012; Yuan et al., 2014) and are also consistent with qualitative research findings (Kaddoura, et al., 2016). In Yuan et al.'s (2014) study, 113 s- and third-year nursing students showed significantly improved clinical judgment scores. Kaddoura (2016), using repeated HFS sessions with 107 first-year nursing students, discovered enhanced critical thinking, competence, and confidence. Price et al. (2017), employing experimental design with novice nurses, observed participants utilizing both analytical and intuitive approaches in their decision-making. Narrowed thinking, the lack of using methodical approaches and considering limited options may contribute to reducing practitioner's effectiveness in solving clinical problems as demonstrated (Croskerry et al., 2013a). This was also consistent with the first phase of our study that found delays in students identifying and managing a clinical problem when they used typical presentation for cues interpretation or routine practice that was inapplicable to the presented cues (Abdulmohdi & McVicar, 2022).

Students referred to an easy grasp of the situation that leads to quick impulsive decisions, but they became more self-aware of the need to mitigate this limitation by seeking more information before executing quick decisions. They described how in their clinical practice they are aware of the different types of decision-making and recognize the importance of validating their first impression. This resonates with the dual process theory description of people using two types of CDM that override each other if one type does not adequately solve a problem (Croskerry & Nimmo, 2011). The nonanalytical approach (Type 1, unconscious decisions) refers to the use of intuition or pattern recognition or automated behaviors and the analytical approach of CDM (Type 2, conscious decision) refers mainly to the use of the hypotheticodeductive method. Therefore, simulation could help students learn how to think about their thinking in order to calibrate their cognitive processing. This supports the findings from the “think aloud” data in phase one of the study, which identified students using both types of decision-making and employing cognitive biases in problem-solving (Abdulmohdi & McVicar, 2022). It also aligns with the results of other studies (Price et al., 2017; Thompson et al., 2009). Price (2017) employed experimental design among novice nurses and found that participants used both analytical and intuition.

The findings of this study demonstrated that clinical simulation has increased students’ awareness of cognitive biases. This awareness led them to improve their approach in thinking logically and more systematically to mitigate and regulate these biases. Students also discussed how debriefing was important for them to develop self-awareness about their errors and areas of development. Croskerry et al. (2013b) recommended to use strategies to increase people’s awareness about their biases as the first step to regular and avoid future biases.

Overall, going through the simulation learning experience helped students to think more about how they might improve their skills and to perform tasks differently and more effectively in the future, a fundamental part of the reflective process. Linking this to Kolb's experiential learning theory cycle (Kolb, 2015) it could suggest that they are moving from Stage 2 (reflective observation) into Stage 3 (abstract conceptualization) as they concluded their learning from their experience and aimed to change the way they practice. According to Kolb (2015), people do not learn just by reviewing but also by doing and then critically evaluating what they did through reflective observation. The outcome is to make sense of what has happened and move to a final stage (Stage 4) to put what they learnt into their practice.

Students perceived the simulation session as a useful experience to prepare for the real world of practice and promoted a change in their behaviors about how to process information and make decisions. They recalled what happened during the simulation session in their clinical placement. Likewise, Hustad et al. (2019) found that HFS mentally prepared students for their clinical practice. During the simulated learning students in this study applied the ABCDE approach to structure their information gathering, processing, and making sense of the situation. They described using this method more effectively when approaching an actual clinical situation in practice. Students also discussed how the simulated experience helped them optimize their performance, avoid errors in their clinical practice and gain more clinical confidence.

Students’ descriptions demonstrate their ability to access and utilize knowledge learnt in simulation in clinical situations and evidence the transferability of learning. This is consistent with a few studies where students perceived the benefits of simulation in preparing them to practice (Botma, 2014; Liaw et al., 2012), and integrating their learning from the simulation into practice (Botma, 2014; Kaddoura, 2016). They emphasized the authenticity of HFS, which closely mirrored their real-world clinical experiences, particularly when dealing with complex comorbidities. Students described they felt similar pressure and a sense of responsibility during the simulated experience like how they sometimes feel in their clinical placement. The similarity between how the knowledge was originally learning, and how it was retrieved, has been described to facilitate information recall and the application of theory to practice (Eva et al., 1998). Sweller (1988) reported that dependent on the level of experience and the complexity of a task, a person could maintain their focus on specific parts of a given situation during performance due to the limited capacity of the working memory. Repetitive and incremental individualized simulated sessions could be beneficial for learners as they aim to enhance their information processing capacity, particularly as they accumulate more clinical experience.

### Strengths and Limitations

This study collected subjective data from final-year nursing students who attended an HFS session. A strength of this study is being part of a multiphase study, and the findings from this follow-up phase in clinical placement validated the results from the experimental phase one in simulation settings, adding more credibility to the benefits of simulation obtained through multiple methods. The participants were from one university and the study involved convenience sampling with students who voluntarily participated. Therefore, our findings from this study are not generalizable. Nevertheless, the results could provide valid data that support the role of HFS in developing clinical decision-making skills and regulating cognitive biases.

### Implication for Practice

Designing simulation activities based on the theory of clinical decision-making increases the development of decision-making skills for practice and enhances simulation effectiveness. Additionally, structured debriefing and self-evaluation are crucial for students’ self-awareness of their abilities and biases, behavior regulation, and confidence improvement, highlighting the need for ongoing integration and reflective practices in nursing education.

## Conclusion

The present study reports on students’ perceptions of the learning processes that occurred during the simulation and the transferability of that learning to clinical practice. The study highlights that simulation exercises enhance nursing students’ ability to apply theory to practice, improving their decision-making skills. These findings offer valuable insights for enhancing nursing curricula and ensuring effective knowledge transfer to clinical care settings. A higher degree of simulation fidelity and the perceived authenticity of activities in relation to real-world scenarios lead to increased transferability and application of acquired knowledge. Increasing learners’ active engagement in simulated activities could also contribute to enhancing the transfer of learning to clinical practice.
